# Appeasement function of displacement behaviours? Dogs’ behavioural displays exhibited towards threatening and neutral humans

**DOI:** 10.1007/s10071-023-01742-9

**Published:** 2023-01-20

**Authors:** Giulia Pedretti, Chiara Canori, Eleonora Biffi, Sarah Marshall-Pescini, Paola Valsecchi

**Affiliations:** 1 Department of Medicine and Surgery, University of Parma, Via Gramsci 14, 43126 Parma, Italy; 2 Department of Chemistry, Life Science and Environmental Sustainability, University of Parma, Viale delle Scienze 17/A, 43124 Parma, Italy; 3 Domestication Lab, Wolf Science Center, Konrad-Lorenz-Institute for Ethology, University of Veterinary Medicine, Veterinärplatz 1, 1210 Vienna, Austria

**Keywords:** Displacement behaviours, Appeasement signals, Domestic dog, Visual communication

## Abstract

Appeasement signals are behavioural patterns displaying an animal’s non-aggressive attitude and are hypothesized to reduce the aggressive behaviours in the receiver. In domestic dogs, specific displacement behaviours (i.e., behavioural patterns exhibited without an apparent function related to the ongoing situation), have been suggested to function as appeasement signals. To test this possibility, we assessed whether the occurrence of these behaviours was dependent on a social conflict context, predicting that, if displacement behaviours also function as appeasement signals, they should be more prevalent in a conflict vs. non-conflict context. Fifty-three dogs were exposed to two unfamiliar humans approaching them in either a mildly threatening or neutral way. We categorized the attitude of the dogs towards the strangers as “reactive”, i.e., barking and lunging towards the stimulus, and “non-reactive”, i.e., remaining passive in front of the stimuli. We coded dogs’ displacement activities and modelled their duration or frequency as a function of the interaction between the test condition and the attitude of the dog. Displacement behaviours of “blinking”, “nose licking” and “lip wiping” were associated with a “non-reactive” attitude, independently from the test condition, confirming an association with a non-aggressive intention. “Head turning” was associated with a “non-reactive” attitude in the threatening condition. In conclusion, dogs with a non-aggressive attitude exhibited more putative appeasement signals; however, these were not strictly associated with a conflict-ridden situation, calling for further investigation of their function.

**Supplementary Information:**

The online version contains supplementary material available at 10.1007/s10071-023-01742-9.

## Introduction

Displacement activities are behavioural patterns displayed without an apparent function related to the ongoing situation (Maestripieri et al. 1992; Zeigler 1964). They include self-directed behaviours such as scratching, lips licking, yawning, blinking, auto-grooming and environment-directed activities such as sniffing the environment. Characterized as indicators of motivational conflict and a potential by-product of a physiological stress response (Delius 1967; Troisi 2002) (in primates: Schino et al. 1996; in dogs: Beerda et al. 2000; Beerda et al. 1997), these behaviours could also transmit useful information to social partners, indicating the signaler’s discomfort related to the ongoing situation, and may have been selected as visual communicative signals (Bradshaw 1993; Whitehouse et al. 2016). For example, macaques are less likely to attack conspecifics exhibiting displacement behaviours (Whitehouse et al. 2017). Communicative signals are displays exhibited by a sender, evolved to change the behaviour of another individual, the receiver (Laidre and Johnstone 2013). Signals may evolve from precursor behaviours, originally without a communicative function, through a process of ritualization (Maglieri et al. 2022). Precursor behaviours can be intentional movements but also activities linked with motivational conflict, such as displacement behaviours (Tinbergen 1952; Weible 2012).

In domestic dogs, many displacement behaviours have been suggested to function as appeasement signals in dog–dog as well as dog–human interactions (see Overall 2017—for a critical analysis) (see Table 1). Appeasement signals are exhibited by an animal during a conflict-ridden situation communicating its non-aggressive attitude. These signals are hypothesized to have a de-escalation function, interrupting or preventing the aggressive interaction between opponents (Kuhne et al. 2014; Pastore et al. 2011). To date the evidence supporting the hypothesis that displacement behaviours in dogs may function as appeasement signals are scarce.

**Table 1:** List of domestic dogs’ displacement behaviours and references to the literature categorizing them as displacement behaviours/stress indicators or appeasement signals

Displacement behaviours	Classified as displacement behaviours/stress indicators	Classified as appeasement signals
Lips licking	Pastore et al. (2011); Landsberg et al. (2011); Cafazzo et al. (2014)	Rugaas (2006); Pastore et al. (2011); Firnkes et al. (2017)
Nose licking	Väisänen et al. (2005)	Rugaas (2006); Mariti et al. (2014)
Paw lifting	Väisänen et al. (2005); Pastore et al. (2011)	Rugaas (2006); Kuhne et al. (2014); Mariti et al. (2014)
Yawning	Handelman (2012); Pastore et al. (2011); Landsberg et al. (2011; Cafazzo et al. (2014); Howell and Feyrecilde (2018); Townsend and Gee (2021)	Rugaas (2006); Aloff (2005)
Head turning	Pastore et al. (2011)	Rugaas (2006); Mariti et al. (2014); Kuhne et al. (2014)
Sniffing	Aloff (2005); Handelman (2012); Howell and Feyrecilde (2018); Townsend and Gee (2021)	Rugaas (2006); Aloff (2005); Mariti et al. (2014)
Autogrooming and scratching (often coded as unique behaviour)	Handelman (2012); Aloff (2005); Väisänen et al. (2005); Spangenberg et al. (2006); Kuhne et al. (2012); Landsberg et al. (2011); Cafazzo et al. (2014); Howell and Feyrecilde (2018)	Rugaas (2006); Aloff (2005)
Stretching	Väisänen et al. (2005); Spangenberg et al. (2006); Kuhne et al. (2012); Kuhne et al. (2014)	
Shaking	Handelman (2012); Kuhne et al. (2012); Kuhne et al. (2014)	Rugaas (2006)
Blinking^a^	Handelman (2012); Bremhorst et al. (2019) (considered as stress/frustration signal)	Rugaas (2006); Kuhne et al. (2014); Mariti et al. (2017); Siniscalchi et al. (2018);

^a^Blink does not appear in the literature as a displacement behaviour in dogs but has been considered a stress and frustration indicator and is analogous in its nature to self-directed displacement behaviours (e.g., scratching, nose lick)

At the intraspecific level, a single study systematically investigated whether the performance of certain displacement behaviours could also have a communicative, specifically appeasement, function (Mariti et al. 2017). Twenty-four dogs (senders) were observed while interacting with conspecifics (receivers) of different sexes and levels of familiarity (familiar/unfamiliar, female/male), and the display of putative appeasement signals as well as aggressive behaviours was measured. Putative appeasement signals were observed more often when dogs were interacting in close proximity with one another, suggesting a socially relevant valence of these behaviours. Furthermore, the displacement behaviours of head turning, nose licking, and paw lifting were displayed more frequently when dogs were interacting with unfamiliar conspecifics. Aggressive interactions were never preceded by a putative appeasement signal and, if these signals were displayed after an aggression (compared to not being displayed), it was less likely that the intensity of the aggression increased. The authors suggested a possible communicative function of these behaviours as de-escalation and prevention of aggression. However, Mariti et al. (2017) included both displacement behaviours (sniffing the ground, yawning and paw lifting, nose licking) and other putative appeasement behaviours (turning away, slow movements, play bow, lying down, curving, low wagging, crouching) into a single category for the analyses, limiting our understanding of the potential differences in the use of the single behaviours.

Firnkes et al. (2017), instead, focused on the emission of two displacement behaviours (lips licking and head turning) by dogs exposed to nineteen test situations including a human approaching them in a friendly or threatening way, as well as different environmental stressors. Results showed that the behaviour of “head turning” was observed more in the threatening staring and screaming compared to the physical threat and the friendly salutation. “Lips licking" was more frequent as a reaction to the threatening staring and friendly salutation tests compared to the threatening screaming and physical threat. Furthermore, in contact situations, lips licking was more frequently associated with active submission behaviours (ears flattened, tail wagging, crouched posture) compared to socio-positive behaviours (neutral posture, cocked ears, raised tail, eye contact), suggesting an association with a reduced distance between social partners and with a submissive/docile attitude of the dogs. However, the exposure to nineteen stressful test conditions always in the same order makes it difficult to disentangle whether these behavioural patterns were elicited by the specific context, or by the overall stressful situation.

The Threatening Approach Test (TAT) is a widely used paradigm to study dogs’ responsiveness to different human behavioural cues. It consists of exposing dogs to humans approaching them with different attitudes, for example, threatening or friendly/neutral. The TAT has been used to study coping styles in police dogs (Horváth et al. 2007), aggressive reactions in shelter dogs (Kis et al. 2014) and the secure base effect of the attachment of dogs towards their owners (Gácsi et al. 2013). It has been validated by Vas et al. (2005) who showed that dogs change their behavioural response towards a human depending on the latter’s way of approaching. Specifically, dogs were approached by a woman consecutively showing cues of friendliness (calling the dog by its name, normal speed of walk, speaking in a friendly manner) and threat (moving slowly towards the dog with bent upper body looking steadily into the eyes of the dog without speaking). In the friendly approach, most dogs showed “friendly” or “passive” behaviours, while in the threatening approach more than half of them avoided the interaction (“passive avoidant”) or reacted with “threatening” behaviours. Furthermore, a follow-up study showed the consistency of the behavioural reactions of dogs when confronted with different unfamiliar people and when the test was repeated 1 year later (Vas et al. 2008). Dogs reacted consistently, according to the different approaches, independently from the order of exposure.

The TAT is a promising paradigm to study putative appeasement signals in dogs, since it reproduces an ‘ecologically’ valid (a person approaching the dog, while it is being held on the leash by its owner) conflict vs. non-conflict-ridden situation. In this study we aimed to investigate whether displacement behaviours previously identified as putative appeasement signals in dogs are context specific, thus being exhibited more in a conflict-ridden vs. neutral situation and whether they are associated with dog’s different attitudes towards the approaching human (threatening/offensive vs. non-threatening/peaceful).

To answer these research questions, we exposed dogs to a TAT. We adopted a within subject design, where dogs, held on the leash by their owners, were approached sequentially by two experimenters displaying, respectively, a threatening or a neutral approach (counterbalanced order of approaches across subjects). A complete ethogram including both dogs’ facial and non-facial displacement behaviours was adopted for the following analysis.

The appeasement signals hypothesis suggests that displacement behaviours function to reduce the likelihood (or escalation) of conflicts. Thus, they should occur more in potentially conflictual situations, which require avoidance of aggression, compared to neutral situations. Given this, we expect dogs to perform more putative appeasement signals when approached by a threatening compared to a neutral human.

However, based on results from previous studies (Gácsi et al. 2013; Vas et al. 2005), where dogs were observed to individually vary in their reaction to the approaching human, we categorized dogs who reacted with offensive behaviours (barking and lunging towards the stimulus) as “reactive” and dogs who did not display threatening behaviours towards the approaching human as “non-reactive”. We reasoned that since appeasement behaviours should be associated with de-escalation of the conflict, these behaviours should be associated more with “non-reactive” attitude in the tested dog, i.e., with dogs that are not inclined to enter in a conflict situation with the approaching human.

## Materials and methods

### Ethical statement

All the procedures were approved by the ethical committee of the University of Parma (approval numbers PROT. N. 6/CESA/2022). The owners were informed about the experimental procedure and signed a consent form.

### Subjects

Fifty-three domestic dogs were tested, 26 males (intact = 21 and neutered = 5) and 27 females (intact = 8 and neutered = 19). All subjects were adults (aged between 1 and 12 years, mean = 4.45 ± 2.85). Medium to large sized purebred and mixed-breed pet dogs were recruited (see Table 1—Supplemental material for further details). Subjects were recruited from a database of the University of Parma and from clients of two dog training centers, the Green Dog Club (located in Libido San Giacomo—MI) and Ca’Nina (Manerba del Garda—BS). Prior to participation in this study, the dog owners confirmed that their dogs were comfortable with unfamiliar people approaching them.

### Experimental design and set up

The experiments were conducted in three comparable open field areas, between June 2021 and July 2021, at the University of Parma and at the two dog training centers reported above (Green Dog Club and CaNina). For ease of presentation, data from dog–dog approaches will be treated in a separate paper.

**Fig. 1:**
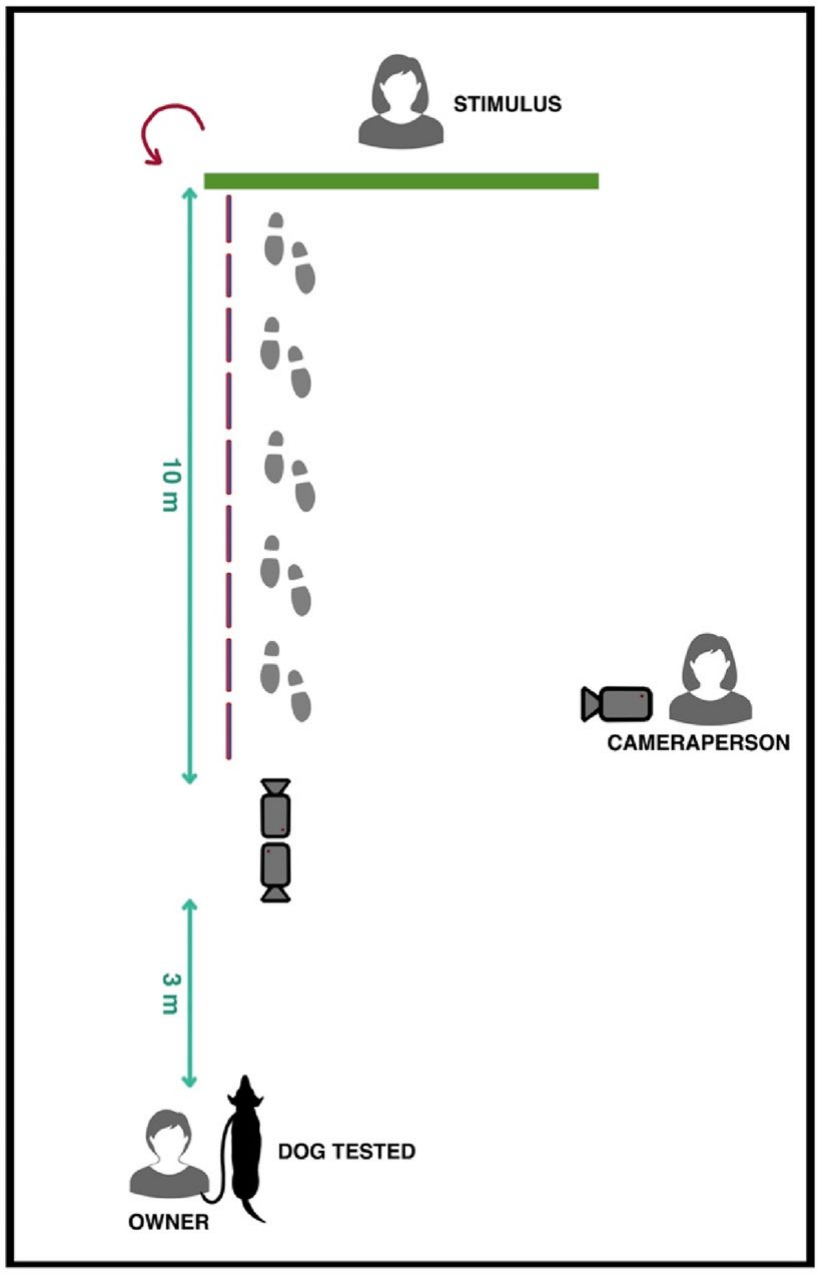
Setup of the experiment

We adopted a within subject design in which each dog was exposed to 2-test stimuli in a counterbalanced way. Two green screens of 1.50 m × 1.50 m were used as an experimental apparatus to hide the stimuli. From the green screen, a 10 m red plastic line was placed on the ground to indicate the stimulus path. At the end of the red line, two fixed cameras were placed to frame both the stimulus and the tested dog. Another camera, held by an experimenter, was used to record all the experimental arena. The owner holding the dog stood three meters from the camera (Fig. 1). The dog wore an H-shaped harness and was held by a 2 m leash.

### Experimental procedure

The duration of the entire procedure was about 15 min. The dog was exposed to two different unfamiliar experimenters approaching it in, respectively, a threatening and a neutral manner. During the test a third person, the cameraperson, remained stationary in the same position filming the test (see Fig. 1). The owner with their dog arrived at the open field area and was asked to walk around the area for 5 min to get the dog used to the environment. In the meantime, the first experimenter hid behind one of the two green screens. The owner was asked to wear a facial mask and sunglasses to avoid any involuntary cuing of the dog. After the walk, the owner was asked to position themselves and the dog in front of the camera. When the owner was in position, the cameraperson gave the “start” signal, and the first experimental condition took place. After the end of the first session, the owners and their dog walked for a few minutes around the area and were then asked to stand in front of the second screen, where the second experimental condition took place.

The two experimental conditions consisted of:

- Threatening Human (TH): at the start of the session, the experimenter, hidden behind the green screen, whistled to get the dog's attention, and then walked outside the green screen along the red line towards the owner/dog dyad (see Fig. 1). The experimenter moved slowly staring the dog in the eyes with a bent body posture and an “angry expression”, wrinkling the forehead (dogs have been shown to be sensitive to humans’ happy/angry expressions—Albuquerque et al. 2016, 2018). The experimenter walked for 10 s and then stopped 3 m from the owner and the dog, maintaining the same facial expression and position of the body and standing still for 20 s.
- Neutral Human (NH): at the start of the session, the experimenter, hidden behind the green screen, whistled to get the dog's attention, and then walked outside the green screen along the red line towards the owner/dog dyad (see Fig. 1). The experimenter walked at a normal speed, with an erected and relaxed body posture, smiling and alternating a soft look at the dog and then averting the gaze. The experimenter walked for 10 s and then, maintaining the same facial expression, stood still for 20 s in the same location described above.

For this study, 8 different experimenters were used as stimuli. To avoid the influence of the stimulus’ sex (Hennessy et al. 1998), only female experimenters were involved. The order of presentation of the conditions was randomly assigned and counterbalanced across subjects.

### Behavioural coding

Dog’s facial expressions and behavioural displays were recorded with three different cameras. The program Shotcut (https://shotcut.org/), a cross-platform video editor, was used to combine the three videos. The combined videos were then analyzed using Solomon Coder Beta 15.01.2013 (Andrá Péter, http://solomoncoder.com).

An ethogram including both facial actions (e.g., “blinking”, “nose licking”, “lip wiping”, “tongue flicking”) and general displacement behaviours was redacted (see Table 2 of the supplemental material for the list of all behavioural variables coded and their respective operational definition).

Videos were coded by two different experimenters (GP and EB), who analyzed 22 videos (10% of the videos of all the procedures) to assess the inter-rater reliability. Intra-class correlations (ICCs; Rousson 2011) were performed in R 4.1.0 (function: ICC; package: psych). Displacement behaviours showed a good reliability ICC = 0.80.

**Fig. 2:**
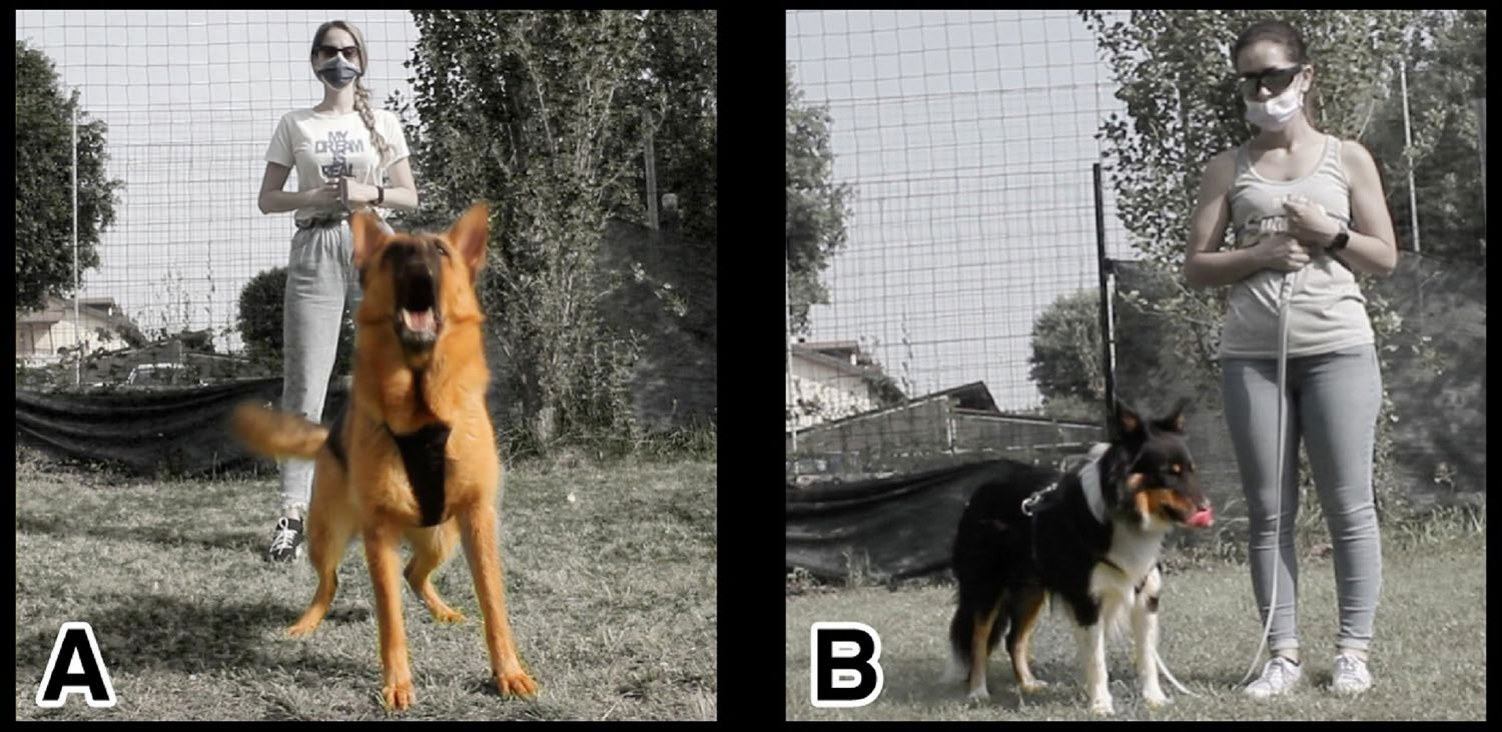
Picture of a “reactive” (**A**) and a “non-reactive” (**B**) attitude

A clear distinction appeared in dogs’ attitudes towards the experimenters during both human approaches with some dogs exhibiting active offensive/defensive behaviours, while other dogs remaining passive/non-offensive. Thus, if the dog exhibited the behaviour of barking or growling towards the experimenter was classified as “reactive”, in contrast, if the dog did not display bark and/or growl towards the experimenter it was classified as “non-reactive” (Fig. 2).

### Statistical analysis

Only displacement behaviours expressed by more than 10% of the dogs in at least one of the two test conditions were considered for further statistical analysis (see Table 5—Supplemental Material).To assess whether the attitude of the dogs (“reactive”/”non-reactive”) was dependent on the condition (threatening or neutral approach) we ran a Generalized Linear Model using the function “glm” with the dogs attitude (binomial response “reactive/non-reactive”) as the response variable, the condition (TH/NH) as fixed factor and including the age, sex, order of exposure and area as control factors.

To assess the effect of the condition (neutral or threatening approach) and the dog’s attitude (“reactive”/“non-reactive”) on the behavioural variables exhibited by dogs, we used Generalized Linear Mixed Models (GLMM; Baayen et al. 2008) using the function “glmmTMB” of the package “glmmTMB”. We used the duration or the frequency of each displacement behaviour as response variable. Gaussian error structure was adopted for duration variables while Poisson error structure for frequency variables. We included as fixed effect the interaction between the condition (“neutral human”/“threatening human”) and the attitude of the dog (“reactive”/“non-reactive”). In all the models, we included as control fixed effects the age, the sex of the subject, the area where the test was conducted and the order of exposure of the two conditions. Subject ID was included as random effect in all the models to account for repeated observations of the same individual. For all the models, as an overall test of the impact of the fixed effects and to avoid “cryptic multiple testing” (Forstmeier and Schielzeth 2011), we compared the full model as ascribed above with respective null models lacking the two main fixed factor (“condition” and “attitude”) and their interaction. If the interaction between the condition and the attitude was not significant, a second model with the two fixed effect (“condition” and “attitude”) but without the interaction was considered as the full model. For each behavioural variable, we tested the effect of individual fixed effects of interest (“condition” and “attitude”) by comparing the full model with reduced models lacking them one at a time (Barr et al. 2013). For each full–null model comparison we utilized a likelihood ratio test (Dobson 2002). We checked model stability by dropping individuals one at a time from the data set and comparing the estimates derived for models fitted to these subsets with those obtained for the full data set. These revealed the models to be of acceptable stability (see Tables from 6 to 25—Supplemental Materials). Collinearity was assessed using the function “vif” of the package car (version 3.0-0), applied to the model lacking the random effect. It revealed no higher values than 1.023. For Poisson models overdispersion was checked with the function “check_overdispersion” (Gelman and Hill 2006). Poisson models were not over dispersed (overdispersion ratio < 1.210).

All statistical analyses were performed in R (version 3.6.1; R Core Team 2018).

Results were considered statistically significant if *p* ≤ 0.05.

## Results

Twenty-six out of 53 dogs (49%) had a “reactive” attitude during the TH, while 11 out of 53 (21%) dogs had a “reactive” attitude during the NH. Nine out of 53 dogs (17%) had a “reactive” attitude in both test conditions, thus only 2 (4%) had a “reactive” attitude only in the neutral condition. The attitude of the dog was influenced by the test condition (full–null model comparison: ${x}^{2}$ = 11.723; *df* = 1; *p* = 0.001) with the occurrence of a “reactive” attitude being higher in the TH condition compared to the NH condition (Es = 1.628 ± 0.506, *z* value = 3.220, 95% CI [0.677, 2.675). The order of the test conditions did not influence the attitude of the dogs towards the stimuli (full–null model comparison: ${x}^{2}$ = 3.077; *df* = 1; *p* = 0.079), while the area had an impact on the attitude of the dogs (full–null model comparison: ${x}^{2}$=13.471; *df* = 2; *p* = 0.001).

### Displacement behaviours

A number of displacement behaviours, i.e., autogrooming, shaking, stretching, and yawning were performed only by a few dogs (from 0 to 4) not allowing for meaningful statistical analysis to be carried out on these variables (see Supplemental Material—Table 3 for the percentages of dogs expressing each behaviour coded in each condition). The displacement behaviours performed by more than 10% of the dogs in at least one of the two test condition were blinking (TH = 64.2%, NH = 87%), head turning (TH = 81%, NH = 94%), paw lifting (TH = 13%, NH = 4), scratching (TH = 1%, NH = 11.3%), nose licking (TH = 34%, NH = 37.7%), lip wiping (TH = 17%, NH = 18.9%) and sniffing the environment (TH = 21%, NH = 39.6%).

Three displacement behaviours were expressed at different rates depending on the dogs’ attitude: a “non-reactive” attitude was characterized by a higher frequency of blinking (“reactive” attitude: mean = 2.914 ± 0.654 and “non-reactive” attitude: mean = 3.507 ± 0.325; full–null model comparison: ${x}^{2}$ = 7.387; *df* = 1; *p* = 0.007), nose licking (“reactive” attitude: mean = 0.257 ± 0.149 and “non-reactive” attitude: mean = 0.788 ± 0.142; full–null model comparison: ${x}^{2}$ = 6.161; *df* = 1; *p* = 0.013) and lip wiping (“reactive” attitude: mean = 0.056 ± 0.055 and “non-reactive” attitude: mean = 0.314 ± 0.069; full–null model comparison: ${x}^{2}$ = 4.572; *df* = 1; *p* = 0.033) (see Table 2 and Fig. 1—Supplemental Material).

**Table 2:** Results of the Generalized linear mixed models regarding the influence of the attitude (“reactive”/”non-reactive”) or the condition (“neutral human”/“threatening human”) on the frequency (*f*) or duration (*d*) of each displacement behaviour

Displacement behaviours	“Reactive” attitude compared to “non-reactive” attitude		
	Estimates	*z* value	95%
Blinking (*f*)	− 0.513 ± 0.197	− 2.605	− 0.860 to − 0.117
Nose licking (*f*)	− 1.048 ± 0.441	− 2.379	− 2.212 to − 0.196
Lip wiping (*f*)	− 1.427 ± 0.779	− 1.831	− 24.878 to − 0.291

	“Neutral human” condition compared to the “threatening human” condition		
Paw lifting (*d*)	0.112 ± 0.051	2.189	0.010 to 0.206

For the results of the other fixed effects on each variable see Supplemental Material Tables from 5 to 12

**Fig. 3:**
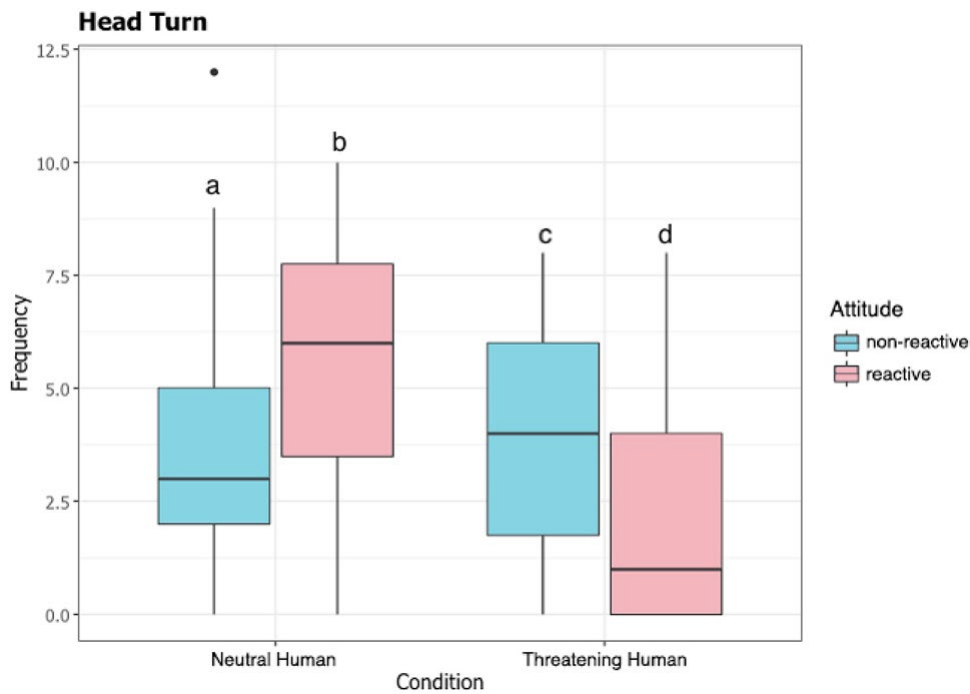
Boxplot of the frequency of performance of head turn as a function of the test condition and attitude of the dogs, (a) Non-reactive dogs in the Neutral human condition, (b) Reactive dogs in the Neutral human condition, (c) Non-reactive dogs in the Threatening human condition, (d) Reactive dogs in the Threatening human condition. Hinges represent IQR (inter-quartile range) and bands represent medians

The frequency of head turns as influenced by the interaction between the attitude and the test condition (full–null model comparison: ${x}^{2}$ = 22.335; *df* = 3; *p* = 0.000). During the threatening approach, head turning was displayed more by dogs with a “non-reactive” attitude compared to those with a “reactive” attitude, while during the neutral approach, dogs expressing a “reactive” attitude showed more head turns than dogs with a “non-reactive” attitude (see Fig. 3).

Finally, the only displacement behaviour influenced by the condition, regardless of the dogs’ attitude was paw lifting (NH: mean = 0.023 ± 0.017 and TH: mean = 0.124 ± 0.017; full–null model comparison: ${x}^{2}$ = 4.639; *df* = 1; *p* = 0.031) which was expressed for longer in the TH compared to the NH (see Table 2 and Fig. 2—Supplemental Material).

## Discussion

This study aimed to test whether dogs’ displacement behaviours may also function as appeasement signals during social interaction with humans. Given the hypothesized function of conflict prevention of these signals, we predicted that they would be exhibited more in conflict-ridden situations compared to neutral ones. Furthermore, since they should promote a de-escalation of the conflict, we predicted they would be associated more with a non-offensive (rather than an offensive) attitude in the tested dog, i.e., with dogs that are not inclined to enter in a conflict situation with the approaching human. To test this hypothesis, we exposed 53 dogs to two human experimenters approaching them in either a threatening or a neutral manner (Threatening Approach Test—TAT). We categorized the dogs’ attitude as “reactive” if the dog barked and lunged towards the experimenter and “non-reactive” if they did not exhibit these behaviours.

Approximately half of the dogs showed a “reactive” attitude in the threatening human condition and only 21% of dogs in the neutral condition. Although for 17% of dogs both test conditions (TH and NH) may have been perceived as challenging, the threatening condition more frequently elicited a reactive attitude in the tested dogs. Displacement behaviours of “blinking”, “nose licking” and “lip wiping” were positively associated with a “non-reactive” attitude both in the threatening and in the neutral condition. A recent study from our group found that these patterns are exhibited with higher probability in a frustration evoking situation (denial of food reward) when a human partner was visible compared to when it was not visible, suggesting a possible communicative valence of these visual signals (Pedretti et al. 2022). Furthermore, a previous study by Firnkes and colleagues found that lips licking is displayed more frequently in the mild threatening/friendly approach compared to a threatening screaming and physical threat approach (Firnkes et al. 2017) and it was frequently associated with active submissive behaviours (ears flattened, tail wagging, crouched posture), suggesting a potential function in mediating social encounters. Considering results of both studies these signals are meaningful in a social context (Pedretti et al. 2022); however, their function is not limited to a potential conflict situation (Firnkes et al. 2017). The appeasement function is thus not fully confirmed: although these behaviours were exhibited predominantly by dogs that lacked offensive/defensive motivation (“non-reactive” dogs), there was not a higher frequency of such behaviours in the more conflictual vs. neutral context as expected.

Finally, the behaviour of head turning was expressed more when dogs engaged in a non-reactive attitude during the threatening human condition but not in the neutral one. In fact, dogs displaying a “reactive” attitude in the threatening condition looked at the stimulus more compared to dogs displaying a “reactive” attitude in the neutral condition. Early studies suggest that staring and direct eye-contact is a component of offensive threat in dogs’ conspecific interactions (Simpson 1997). Other studies highlight that dogs could interpret the staring of a human as a mild threatening signal (Duranton et al. 2017; Soproni et al. 2001). Present results confirm these hypotheses showing how dogs who had an offensive/defensive reaction towards the threatening stimuli did not perform many head turns and instead stared at the human for longer.

Finally, the only displacement behaviour performed for a longer duration in the threatening compared to the neutral condition, independent of the dogs’ attitudes, was the paw lifting. This behaviour has been identified as an indicator of stress (Beerda et al. 1997; Rooney et al. 2007; Srithunyarat et al. 2017), fear and anxiety (Loftus et al. 2012), and as an appeasement signal as well ( Kuhne et al. 2012; Rugaas 2006). Handelman (2012) suggests paw-lift to be a behaviour indicating uncertainty and warning of an upcoming agonistic behaviour. Current result cannot tease apart these different functions; however, they show that this behaviour is elicited more in a higher intensity conflict situation regardless of the dogs’ attitude.

In conclusion dogs displaying a “non-reactive” attitude (i.e., non-threatening towards the human approaching) expressed more putative “appeasement signals”, confirming their possible link with a non-aggressive attitude, and a motivation to de-escalate a potential conflict. However, in contrast to our prediction, these dogs performed most displacement behaviours (i.e., “nose licking”, “blinking”, “sniffing the environment”) with the same rate of expression in both neutral and threatening approaches. It is worth noting that even though previous studies have shown that dogs can discriminate between smiling and non-smiling human faces (Nagasawa et al. 2011), and can indeed differentiate between the different approaches of the TAT test (Gácsi et al. 2013; Vas et al. 2005), dogs with a “non-reactive” attitude may perceive both conditions as equally ambiguous, thereby performing a series of displacement behaviours in both contexts. Thus, these behaviours may carry a communicative valence linked with non-aggressive intentions (Pedretti et al. 2022); however, their appeasement function cannot be confirmed yet. Indeed, Aloff (2005) suggests that they may be used as a means to manage dogs’ personal space in any social interaction, and not only in a conflict-ridden situation. Further studies should investigate single displacement behaviours in relation to the context, the underlying motivation of the sender and, finally, the feedback of receivers, especially conspecifics, to allow a correct interpretation of their nature and function before including them in a priori categories (i.e., “stress signals”, “appeasement signals”). To this aim, the exhibition of putative appeasement signals should be further investigated in dog–dog interactions using standardized and replicable methods in both behavioural coding and statistical analysis, since it is possible that during dog–human interactions dogs are more prone to use evident threatening/offensive behaviours (barking towards the stimulus) and less subtle appeasement signals that humans may not be able to detect (Mariti et al. 2012). Furthermore, as suggested by Overall (2017), for the function of appeasement signal to be met, the signal must be further studied not only in relation to the specific types of interactions but also to the responses of the receivers and concurrent changes in behaviours of the sender.

## Supplementary Information

Below is the link to the electronic supplementary material.

Supplementary file1 (DOCX 148 KB)

## Data Availability

The raw data and analysis of this study are available from the corresponding author on request.
