# Supplementary material for: Appeasement function of displacement behaviours? Dogs’ behavioural displays exhibited towards threatening and neutral humans
Source: Anim Cogn. 2023 Jan 20;26(3):943–52. doi: 10.1007/s10071-023-01742-9 (PMC10066101; doi:10.1007/s10071-023-01742-9)
Supplement: Supplementary file 1 — Supplementary file1 (DOCX 148 KB) [file 10071_2023_1742_MOESM1_ESM.docx]

*SUPPLEMENTAL MATERIAL*

**Table 1 – SUBJECT INCLUDED IN THE STUDY**

| **Subject** | **Attitude in TH** | **Attitude in NH** | **Sex** | **Age** | **Castration  status** | **Breed** | **Order** |
| --- | --- | --- | --- | --- | --- | --- | --- |
| Aaron | no | yes | m | 5 | no | Appenzeller Sennenhund | TH/NH |
| Alaska | no | no | f | 2 | yes | Mix breed | NH/TH |
| Alaska2 | no | no | m | 3 | no | Lupino del Gigante | TH/NH |
| Altea | yes | no | f | 12 | yes | Labrador Retriever | TH/NH |
| Amali | no | no | f | 11 | yes | Rhodesian Ridgeback | TH/NH |
| Antares | no | no | m | 7 | no | Czezhoslovakian Wolfdog | NH/TH |
| Argo | yes | yes | m | 3 | no | Mix breed | TH/NH |
| Bear | yes | no | m | 3 | yes | Mix breed | NH/TH |
| Blue | yes | yes | m | 3 | no | Golden retriever | NH/TH |
| Bonnie | no | no | f | 4 | yes | Mix breed | TH/NH |
| Boris | yes | yes | m | 2 | no | Australian Shepherd | TH/NH |
| Brioche | no | no | f | 1 | no | Pit bull | TH/NH |
| Chucky | yes | yes | m | 4 | no | Australian Shepherd | NH/TH |
| Clide | no | no | m | 4 | no | Mix breed | TH/NH |
| Cocò | yes | no | f | 3 | no | Labrador Retriever | TH/NH |
| Daisy | no | no | f | 3 | yes | Dobermann | NH/TH |
| Dea | yes | no | f | 2.5 | no | German Shepherd | NH/TH |
| Demon | yes | yes | m | 7 | no | Weimaraner | NH/TH |
| Elsa | no | no | f | 6 | yes | Mix breed | TH/NH |
| Eren | no | no | m | 3 | yes | Husky | TH/NH |
| Grace | yes | yes | f | 5 | yes | Weimaraner | TH/NH |
| Hero | yes | no | f | 7 | yes | Australian Shepherd | TH/NH |
| Iris | no | no | f | 9 | yes | Labrador | NH/TH |
| Kappe | yes | no | m | 2 | no | Australian Shepherd | NH/HT |
| Kira | no | no | f | 5 | yes | Mix breed | TH/NH |
| Lapo | yes | no | m | 8 | yes | Border Collie | NH/TH |
| Leluche | no | no | m | 3 | no | Australian Shepherd | TH/NH |
| Leo | no | no | m | 7 | no | Lagotto Romagnolo | TH/NH |
| Lilo | yes | no | f | 11 | yes | Australian Shepherd | TH/NH |
| Lucky | no | yes | m | 1 | no | Border Collie | NH/TH |
| Lucky2 | no | no | m | 6 | no | Mix breed | TH/NH |
| Luna | yes | no | f | 1 | no | Mix breed | TH/NH |
| Maya | yes | yes | f | 2 | yes | Riesenschnauzer | NH/TH |
| Megghy | no | no | f | 10 | yes | Czezhoslovakian Wolfdog | NH/TH |
| Mulan | yes | no | f | 1 | no | Hovawart | TH/NH |
| Nala | yes | no | f | 7 | yes | Malinois | TH/NH |
| Nanook | yes | no | f | 2 | yes | Australian Shepherd | TH/NH |
| Nelson | yes | no | m | 4 | no | Border Collie | TH/NH |
| Oscar | no | no | m | 4 | yes | Mix breed | TH/NH |
| Oscar2 | yes | no | m | 1 | yes | Mix breed | TH/NH |
| Ottavia | no | no | f | 4 | no | Australian Shepherd | TH/NH |
| Pegghy | no | no | f | 4 | yes | Mix breed | NH/TH |
| pluma | no | no | f | 4 | yes | Amstaff | TH/NH |
| Simba | yes | no | m | 10 | no | Golden Retriever | TH/NH |
| Sky | yes | no | m | 1 | no | Mix breed | NH/TH |
| Spirit | yes | yes | m | 2.5 | no | Golden Retriever | TH/NH |
| Subbuglio | no | no | m | 2 | yes | Mix breed | TH/NH |
| Tessa | no | no | f | 7 | yes | Drahthaar | NH/TH |
| Thanos | no | no | m | 2 | no | Rhodesian Ridgeback | TH/NH |
| Thea | yes | yes | f | 2.5 | yes | Australian Shepherd | TH/NH |
| Wendi | yes | no | f | 4.5 | yes | Mix breed | NH/TH |
| Zeus | no | no | m | 2 | no | Australian Shepherd | TH/NH |
| Ziva | no | no | f | 6.5 | no | Weimaraner | TH/NH |

**Table 2 - DISPLACEMENT BEHAVIOURS ETHOGRAM**

| **General Category** | **Behaviour** | **Description** | **Type of measure** |
| --- | --- | --- | --- |
| Displacement behaviours | Autogrooming | The dog starts licking, wiping or nibbling its own body. | Duration |
|  | Paw lifting | The dog lifts one of the front paws, even slightly. | Duration |
|  | Blinking | Upper and lower eyelids move towards together until they completely touch each other when the eyes close completely. In the blinking **eyes open again within half a second**. | Event |
|  | Scratching | The dog scratches its neck, face or body with the hind legs. | Duration |
|  | Shaking | Dog's whole body and/or head starts moving rapidly, from side to side while the dog stands. | Event |
|  | Stretching | The whole body is stretched in various ways: forelegs may be leant down while the dog's back is arched; hind legs may be straightened while the head is held up high. | Duration |
|  | Yawning | The mouth is wide open, the neck is stretched. | Event |
|  | Nose licking | The dog licks its nose without lateral movements of the tongue on the lips. | Event |
|  | Lip wiping | The mouth is slightly open, and the dog licks its upper lip, on the right or on the left | Event |
|  | Head turning | Dog turns its head or body away from a stimulus. | Event |
|  | Sniffing the environment | The dog sniffs the environment. | Duration |

**Table 3 – DISPLACEMENT BEHAVIOURS**

Percentage of dogs performing behaviours in the Threatening and Neutral human condition.

| **Category** | **Behaviours** | **n/tot (= 53)** | **Threatening human** | **n/tot (= 53)** | **Neutral human** |
| --- | --- | --- | --- | --- | --- |
| **Displacement behaviours** | Blinking | 34 | 64.2% | 46 | 87% |
|  | Head turning | 43 | 81% | 50 | 94.3% |
|  | Paw lifting | 7 | 13% | 2 | 4% |
|  | Scratching | 1 | 2% | 6 | 11.3% |
|  | Nose licking | 18 | 34% | 20 | 37.7% |
|  | Lip wiping | 9 | 17% | 10 | 18.9% |
|  | Sniffing the environment | 11 | 21% | 21 | 39.6% |
|  | Autogrooming | 2 | 4% | 1 | 2% |
|  | Shaking | 0 | 0% | 3 | 6% |
|  | Stretching | 0 | 0% | 1 | 2% |
|  | Yawning | 4 | 8% | 4 | 8% |

**Table 4** - Behaviours selected for statistical analysis, exhibited in at least 10% of the tests. (* displacement behaviours)

| **Ethogram** | **Category** | **Behaviour** |
| --- | --- | --- |
| **Displacement behaviours** |  | *Blinking |
|  |  | *Nose licking |
|  |  | *Lip wiping |
|  |  | *Head turning |
|  |  | *Paw lifting |
|  |  | *Scratching |
|  |  | *Sniffing the environment |

**Figure 1** - Boxplot of the frequency of performance of displacement behaviours as a function of the attitude of the dogs. Hinges represent IQR (inter-quartile range) and bands represent medians.


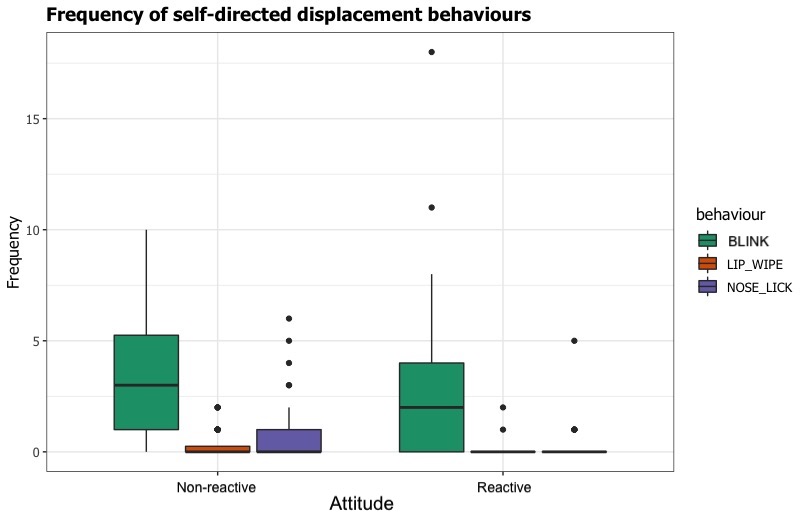


**Figure 2** – Boxplot of the duration of performance of paw lifting as a function of the attitude of the dogs. Hinges represent IQR (inter-quartile range) and bands represent medians.


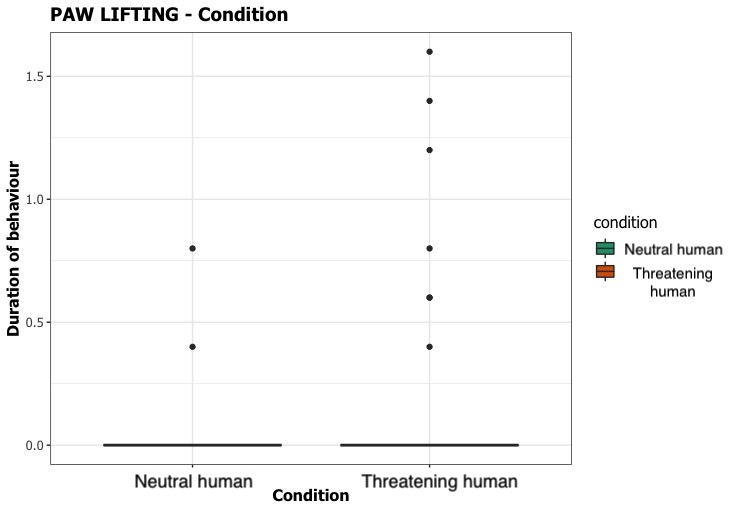


**Results of the statistical models**

**Table 5 –** Effects of condition, order and area on dogs’ attitude (reactive/non-reactive), included as a binomial response.

| **condition** | **Resid. Df** | **Resid. Dev** | **Df** | **Deviance** | **Pr(>Chi)** |
| --- | --- | --- | --- | --- | --- |
| full | 99 | 106.167 | NA | NA | NA |
| null | 100 | 117.890 | -1.000 | -11.723 | 0.001 |

| **order** | **Resid. Df** | **Resid. Dev** | **Df** | **Deviance** | **Pr(>Chi)** |
| --- | --- | --- | --- | --- | --- |
| Null | 94 | 101.147 | NA | NA | NA |
| full | 99 | 106.167 | -5.000 | -5.020 | 0.413 |

| **area** | **Resid. Df** | **Resid. Dev** | **Df** | **Deviance** | **Pr(>Chi)** |
| --- | --- | --- | --- | --- | --- |
| Null | 101 | 119.638 | NA | NA | NA |
| full | 99 | 106.167 | 2.000 | 13.471 | 0.001 |

| **Full model** | **Df** | **Deviance** | **AIC** | **LRT** | **Pr(>Chi)** |
| --- | --- | --- | --- | --- | --- |
| **<none>** | NA | 106.167 | 120.167 | NA | NA |
| **condition** | 1 | 117.890 | 129.890 | 11.723 | 0.001 |
| **age** | 1 | 107.046 | 119.046 | 0.879 | 0.349 |
| **sex** | 1 | 107.875 | 119.875 | 1.708 | 0.191 |
| **order** | 1 | 109.244 | 121.244 | 3.077 | 0.079 |
| **area** | 2 | 119.638 | 129.638 | 13.471 | 0.001 |

| **Full model** | **Estimate** | **Std. Error** | **z-value** | **p-value** | **min** | **max** | **Lower CI** | **Upper CI** |
| --- | --- | --- | --- | --- | --- | --- | --- | --- |
| **(Intercept)** | -2.246 | 0.964 | -2.331 | 0.020 | -2.509 | -1.800 | -4.287 | -0.459 |
| **Condition (Threatening human)** | 1.628 | 0.506 | 3.220 | 0.001 | 1.505 | 1.700 | 0.677 | 2.675 |
| **age** | -0.090 | 0.098 | -0.920 | 0.358 | -0.113 | -0.036 | -0.292 | 0.095 |
| **Sex (male)** | 0.634 | 0.490 | 1.293 | 0.196 | 0.499 | 0.727 | -0.314 | 1.623 |
| **Order (TH_NH)** | -0.896 | 0.517 | -1.733 | 0.083 | -1.038 | -0.745 | -1.940 | 0.105 |
| **Area (greendog)** | 2.276 | 0.777 | 2.930 | 0.003 | 1.910 | 2.472 | 0.871 | 3.981 |
| **Area (parma)** | 0.801 | 0.759 | 1.056 | 0.291 | 0.471 | 1.039 | -0.599 | 2.455 |

Tjur's R2: 0.250

**Table 6** – BLINKING

| **Condition** | **Df** | **AIC** | **BIC** | **logLik** | **deviance** | **Chisq** | **Df** | **Pr(>Chisq)** |
| --- | --- | --- | --- | --- | --- | --- | --- | --- |
| **Null model** | 8 | 506.125 | 527.433 | -245.063 | 490.125 | NA | NA | NA |
| **Full model** | 9 | 505.076 | 529.047 | -243.538 | 487.076 | 3.049 | 1 | 0.081 |

| **Attitude** | **Df** | **AIC** | **BIC** | **logLik** | **deviance** | **Chisq** | **Df** | **Pr(>Chisq)** |
| --- | --- | --- | --- | --- | --- | --- | --- | --- |
| **Null model** | 8 | 510.463 | 531.770 | -247.232 | 494.463 | NA | NA | NA |
| **Full model** | 9 | 505.076 | 529.047 | -243.538 | 487.076 | 7.387 | 1 | 0.007 |

| **FULL MODEL** | **Estimate** | **Std. Error** | **z-value** | **P-value** | **Lower CI** | **Upper CI** | **min** | **max** |
| --- | --- | --- | --- | --- | --- | --- | --- | --- |
| **(Intercept)** | 1.641 | 0.325 | 5.051 | 0.000 | 0.980 | 2.213 | 1.529 | 1.795 |
| **Condition (threatening human)** | -0.207 | 0.119 | -1.743 | 0.081 | -0.447 | 0.006 | -0.307 | -0.147 |
| **Attitude (reactive)** | -0.513 | 0.197 | -2.605 | 0.009 | -0.860 | -0.177 | -0.583 | -0.446 |
| **age** | -0.060 | 0.035 | -1.718 | 0.086 | -0.131 | -0.001 | -0.094 | -0.044 |
| **Sex (male)** | 0.635 | 0.196 | 3.234 | 0.001 | 0.238 | 1.027 | 0.531 | 0.688 |
| **Order (TH_NH)** | -0.416 | 0.200 | -2.076 | 0.038 | -0.816 | -0.013 | -0.488 | -0.273 |
| **Area (greendog)** | -0.298 | 0.268 | -1.111 | 0.266 | -0.836 | 0.279 | -0.424 | -0.128 |
| **Area (parma)** | -0.129 | 0.244 | -0.528 | 5.976 | -0.580 | 0.362 | -0.211 | 0.039 |

Conditional R2: 0.632

Marginal R2: 0.302

**Table 7** – NOSE LICKING

| **Condition** | **Df** | **AIC** | **BIC** | **logLik** | **deviance** | **Chisq** | **Df** | **Pr(>Chisq)** |
| --- | --- | --- | --- | --- | --- | --- | --- | --- |
| **Null model** | 8 | 216.261 | 237.568 | -100.130 | 200.261 | NA | NA | NA |
| **Full model** | 9 | 217.317 | 241.288 | -99.659 | 199.317 | 0.943 | 1 | 0.331 |

| **Attitude** | **Df** | **AIC** | **BIC** | **logLik** | **deviance** | **Chisq** | **Df** | **Pr(>Chisq)** |
| --- | --- | --- | --- | --- | --- | --- | --- | --- |
| **Null model** | 8 | 221.479 | 242.786 | -102.739 | 205.479 | NA | NA | NA |
| **Full model** | 9 | 217.3172 | 241.288 | -99.659 | 199.317 | 6.161 | 1 | 0.013 |

| **FULL MODEL** | **Estimate** | **Std. Error** | **z-value** | **Pr(>\|z\|)** | **Lower CI** | **Upper CI** | **min** | **max** |
| --- | --- | --- | --- | --- | --- | --- | --- | --- |
| **(Intercept)** | -0.830 | 0.674 | -1.231 | 0.218 | -2.394 | 0.405 | -1.237 | -0.301 |
| **Condition (threatening human)** | 0.256 | 0.264 | 0.971 | 0.332 | -0.291 | 0.789 | 0.159 | 0.339 |
| **Attitude (reactive)** | -1.048 | 0.441 | -2.379 | 0.017 | -2.212 | -0.196 | -1.741 | -0.950 |
| **age** | -0.093 | 0.071 | -1.322 | 0.186 | -0.252 | 0.048 | -0.177 | -0.060 |
| **Sex (male)** | 0.680 | 0.376 | 1.807 | 0.071 | -0.047 | 1.554 | 0.461 | 0.870 |
| **Order (TH_NH)** | 0.484 | 0.431 | 1.124 | 0.261 | -0.307 | 1.499 | 0.319 | 0.898 |
| **Area (greendog)** | -0.802 | 0.523 | -1.535 | 0.125 | -1.874 | 0.389 | -1.128 | -0.431 |
| **Area (parma)** | -0.004 | 0.440 | -0.010 | 0.992 | -0.846 | 1.016 | -0.206 | 0.328 |

Conditional R2: 0.486

Marginal R2: 0.272

**Table 8** – LIP WIPING

| **Condition** | **Df** | **AIC** | **BIC** | **logLik** | **deviance** | **Chisq** | **Df** | **Pr(>Chisq)** |
| --- | --- | --- | --- | --- | --- | --- | --- | --- |
| **Null model** | 8 | 110.403 | 131.710 | -47.201 | 94.403 | NA | NA | NA |
| **Full model** | 9 | 112.209 | 136.180 | -47.105 | 94.209 | 0.194 | 1 | 0.660 |

| **Attitude** | **Df** | **AIC** | **BIC** | **logLik** | **deviance** | **Chisq** | **Df** | **Pr(>Chisq)** |
| --- | --- | --- | --- | --- | --- | --- | --- | --- |
| **Null model** | 8 | 114.781 | 136.088 | -49.390 | 98.781 | NA | NA | NA |
| **Full model** | 9 | 112.209 | 136.180 | -47.105 | 94.209 | 4.572 | 1 | 0.033 |

| **FULL MODEL** | **Estimate** | **Std. Error** | **z-value** | **P-value** | **Lower CI** | **Upper CI** | **min** | **max** |
| --- | --- | --- | --- | --- | --- | --- | --- | --- |
| **(Intercept)** | -23.425 | 17437.050 | -0.001 | 0.999 | -48.078 | -21.871 | -47.730 | -22.462 |
| **Condition (threatening human)** | -0.184 | 0.419 | -0.438 | 0.661 | -1.207 | 0.753 | -0.404 | 0.074 |
| **Attitude (reactive)** | -1.427 | 0.779 | -1.831 | 0.067 | -24.878 | -0.291 | -22.193 | -1.183 |
| **age** | -0.006 | 0.093 | -0.062 | 0.950 | -0.255 | 0.178 | -0.055 | 0.030 |
| **Sex (male)** | 0.660 | 0.506 | 1.304 | 0.192 | -0.290 | 1.812 | 0.500 | 1.041 |
| **Order (TH_NH)** | 21.876 | 17437.050 | 0.001 | 0.999 | 20.989 | 40.785 | 21.004 | 46.238 |
| **Area (greendog)** | -0.160 | 0.715 | -0.224 | 0.823 | -1.900 | 1.842 | -0.576 | 0.323 |
| **Area (parma)** | 0.741 | 0.605 | 1.224 | 0.221 | -0.346 | 2.581 | 0.508 | 1.218 |

Conditional R2: 0.989

Marginal R2: 0.988

**Table 9** – HEAD TURNING

| **Interaction** | **Df** | **AIC** | **BIC** | **logLik** | **deviance** | **Chisq** | **Df** | **Pr(>Chisq)** |
| --- | --- | --- | --- | --- | --- | --- | --- | --- |
| **Null model** | 7 | 485.054 | 503.698 | -235.527 | 471.054 | NA | NA | NA |
| **Full model** | 10 | 468.718 | 495.353 | -224.359 | 448.718 | 22.335 | 3 | 0.000 |

| **Full model** | **Df** | **AIC** | **LRT** | **Pr(>Chi)** |
| --- | --- | --- | --- | --- |
| **<none>** | NA | 468.718 | NA | NA |
| **age** | 1 | 468.037 | 1.319 | 0.251 |
| **sex** | 1 | 471.636 | 4.918 | 0.027 |
| **order** | 1 | 468.972 | 2.254 | 0.133 |
| **area** | 2 | 465.321 | 0.603 | 0.740 |
| **condition:attitude** | 1 | 474.642 | 7.924 | 0.005 |

| **FULL MODEL** | **Estimate** | **Std. Error** | **z-value** | **P-value** | **Lower CI** | **Upper CI** | **min** | **max** |
| --- | --- | --- | --- | --- | --- | --- | --- | --- |
| **(Intercept)** | 1.532 | 0.274 | 5.596 | 0.000 | 0.992 | 2.068 | 1.435 | 1.725 |
| **Condition (threatening human)** | -0.057 | 0.133 | -0.430 | 0.667 | -0.329 | 0.190 | -0.112 | -0.011 |
| **Attitude (reactive)** | 0.097 | 0.223 | 0.435 | 0.663 | -0.388 | 0.492 | -0.017 | 0.196 |
| **age** | -0.034 | 0.029 | -1.149 | 0.251 | -0.096 | 0.022 | -0.068 | -0.024 |
| **Sex (male)** | 0.366 | 0.163 | 2.244 | 0.025 | 0.070 | 0.674 | 0.314 | 0.417 |
| **Order (TH_NH)** | -0.253 | 0.168 | -1.508 | 0.132 | -0.562 | 0.081 | -0.337 | -0.176 |
| **Area (greendog)** | -0.162 | 0.223 | -0.726 | 0.468 | -0.617 | 0.302 | -0.248 | -0.068 |
| **Area (parma)** | -0.141 | 0.205 | -0.685 | 0.493 | -0.506 | 0.281 | -0.212 | -0.052 |
| **Condition(threateninghuman): attitude(reactive)** | -0.716 | 0.253 | -2.825 | 0.005 | -1.181 | -0.175 | -0.807 | -0.578 |

Conditional R2: 0.519

Marginal R2: 0.242

**Table 10** – PAW LIFTING

| **Condition** | **Df** | **AIC** | **BIC** | **logLik** | **deviance** | **Chisq** | **Df** | **Pr(>Chisq)** |
| --- | --- | --- | --- | --- | --- | --- | --- | --- |
| **Null model** | 9 | 36.082 | 60.053 | -9.0410 | 18.082 | NA | NA | NA |
| **Full model** | 10 | 33.444 | 60.078 | -6.722 | 13.444 | 4.639 | 1 | 0.031 |

| **Attitude** | **Df** | **AIC** | **BIC** | **logLik** | **deviance** | **Chisq** | **Df** | **Pr(>Chisq)** |
| --- | --- | --- | --- | --- | --- | --- | --- | --- |
| **Null model** | 9 | 31.764 | 55.735 | -6.882 | 13.764 | NA | NA | NA |
| **Full model** | 10 | 33.444 | 60.078 | -6.722 | 13.444 | 0.321 | 1 | 0.571 |

| **FULL MODEL** | **Estimate** | **Std-Error** | **z-value** | **P-value** | **Lower CI** | **Upper CI** | **min** | **max** |
| --- | --- | --- | --- | --- | --- | --- | --- | --- |
| **(Intercept)** | 0.002 | 0.095 | 0.022 | 0.983 | -0.183 | 0.177 | -0.023 | 0.032 |
| **Condition (threatening human)** | 0.112 | 0.051 | 2.189 | 0.029 | 0.010 | 0.206 | 0.079 | 0.122 |
| **Attitude (reactive)** | -0.035 | 0.062 | -0.567 | 0.571 | -0.153 | 0.094 | -0.076 | 0.006 |
| **age** | -0.006 | 0.010 | -0.672 | 0.502 | -0.026 | 0.012 | -0.008 | -0.003 |
| **Sex (male)** | 0.092 | 0.054 | 1.699 | 0.089 | -0.015 | 0.196 | 0.064 | 0.122 |
| **Order (TH_NH)** | -0.037 | 0.057 | -0.647 | 0.517 | -0.147 | 0.087 | -0.059 | 0.010 |
| **Area (greendog)** | 0.043 | 0.076 | 0.566 | 0.572 | -0.106 | 0.189 | -0.001 | 0.063 |
| **Area (parma)** | 0.047 | 0.070 | 0.668 | 0.504 | -0.088 | 0.179 | 0.009 | 0.058 |

Conditional R2: 0.161

Marginal R2: 0.083

**Table 11** – SCRATCHING

| **Condition** | **Df** | **AIC** | **BIC** | **logLik** | **deviance** | **Chisq** | **Df** | **Pr(>Chisq)** |
| --- | --- | --- | --- | --- | --- | --- | --- | --- |
| **Null model** | 9 | 333.594 | 359.539 | -157.797 | 315.594 | NA | NA | NA |
| **Full model** | 10 | 333.579 | 362.407 | -156.790 | 313.579 | 2.015 | 1 | 0.156 |

| **Attitude** | **Df** | **AIC** | **BIC** | **logLik** | **deviance** | **Chisq** | **Df** | **Pr(>Chisq)** |
| --- | --- | --- | --- | --- | --- | --- | --- | --- |
| **Null model** | 9 | 331.583 | 357.528 | -156.792 | 313.583 | NA | NA | NA |
| **Full model** | 10 | 333.579 | 362.407 | -156.790 | 313.579 | 0.004 | 1 | 0.951 |

| **FULL MODEL** | **Estimate** | **Std. Error** | **z-value** | **Pr(>\|z\|)** | **Lower CI** | **Upper CI** | **min** | **max** |
| --- | --- | --- | --- | --- | --- | --- | --- | --- |
| **(Intercept)** | -0.181 | 0.259 | -0.701 | 0.484 | -0.698 | 0.338 | -0.231 | -0.049 |
| **Condition (threatening human)** | -0.209 | 0.147 | -1.426 | 0.154 | -0.478 | 0.071 | -0.255 | -0.126 |
| **Attitude (reactive)** | -0.010 | 0.172 | -0.061 | 0.952 | -0.349 | 0.310 | -0.141 | 0.040 |
| **Sex (male)** | 0.039 | 0.144 | 0.271 | 0.786 | -0.248 | 0.333 | -0.030 | 0.131 |
| **age** | 0.048 | 0.027 | 1.785 | 0.074 | -0.002 | 0.103 | 0.025 | 0.057 |
| **Order (TH_NH)** | 0.068 | 0.152 | 0.446 | 0.655 | -0.248 | 0.390 | 0.001 | 0.169 |
| **areagreendog** | 0.463 | 0.212 | 2.178 | 0.029 | 0.047 | 0.889 | 0.312 | 0.532 |
| **areaparma** | 0.075 | 0.188 | 0.398 | 0.691 | -0.281 | 0.465 | 0.028 | 0.108 |

Conditional R2: 0.093

Marginal R2: 0.089

**Table 12** – SNIFFING THE ENVIRONMENT

| **Condition** | **Df** | **AIC** | **BIC** | **logLik** | **deviance** | **Chisq** | **Df** | **Pr(>Chisq)** |
| --- | --- | --- | --- | --- | --- | --- | --- | --- |
| **Null model** | 9 | 608.770 | 632.741 | -295.385 | 590.770 | NA | NA | NA |
| **Full model** | 10 | 610.009 | 636.644 | -295.005 | 590.009 | 0.761 | 1 | 0.383 |

| **Attitude** | **Df** | **AIC** | **BIC** | **logLik** | **deviance** | **Chisq** | **Df** | **Pr(>Chisq)** |
| --- | --- | --- | --- | --- | --- | --- | --- | --- |
| **Null model** | 9 | 610.902 | 634.873 | -296.451 | 592.902 | NA | NA | NA |
| **Full model** | 10 | 610.009 | 636.644 | -295.005 | 590.009 | 2.893 | 1 | 0.089 |

| **FULL MODEL** | **Estimate** | **Std. Error** | **z-value** | **P-value** | **Lower CI** | **Upper CI** | **min** | **max** |
| --- | --- | --- | --- | --- | --- | --- | --- | --- |
| **(Intercept)** | 1.507 | 1.474 | 1.022 | 0.307 | -1.323 | 4.408 | 0.646 | 1.961 |
| **Condition  (threatening human)** | -0.665 | 0.761 | -0.874 | 0.382 | -2.221 | 0.788 | -1.086 | -0.301 |
| **Attitude (reactive)** | -1.610 | 0.941 | -1.710 | 0.087 | -3.473 | 0.122 | -1.744 | -0.982 |
| **age** | 0.038 | 0.149 | 0.258 | 0.796 | -0.262 | 0.326 | 0.002 | 0.081 |
| **Sex (male)** | 0.206 | 0.840 | 0.245 | 0.806 | -1.447 | 1.905 | -0.343 | 0.556 |
| **Order (TH_NH)** | 0.261 | 0.888 | 0.294 | 0.769 | -1.429 | 1.982 | -0.137 | 0.697 |
| **Area (greendog)** | 0.279 | 1.181 | 0.237 | 0.813 | -1.955 | 2.571 | -0.159 | 1.101 |
| **Area (parma)** | 1.109 | 1.092 | 1.016 | 0.310 | -0.986 | 3.220 | 0.373 | 1.881 |

Conditional R2: 0.188

Marginal R2: 0.070
